# Cell Penetrating Peptides, Novel Vectors for Gene Therapy

**DOI:** 10.3390/pharmaceutics12030225

**Published:** 2020-03-03

**Authors:** Rebecca E. Taylor, Maliha Zahid

**Affiliations:** 1 Mechanical Engineering, Biomedical Engineering and Electrical and Computer Engineering, Carnegie Mellon University, Pittsburgh, PA 15213, USA; bex@andrew.cmu.edu; 2 Department of Developmental Biology, University of Pittsburgh School of Medicine, Pittsburgh, PA 15201, USA

**Keywords:** cell penetrating peptides, protein transduction domains, gene therapy, small interfering RNA

## Abstract

Cell penetrating peptides (CPPs), also known as protein transduction domains (PTDs), first identified ~25 years ago, are small, 6–30 amino acid long, synthetic, or naturally occurring peptides, able to carry variety of cargoes across the cellular membranes in an intact, functional form. Since their initial description and characterization, the field of cell penetrating peptides as vectors has exploded. The cargoes they can deliver range from other small peptides, full-length proteins, nucleic acids including RNA and DNA, liposomes, nanoparticles, and viral particles as well as radioisotopes and other fluorescent probes for imaging purposes. In this review, we will focus briefly on their history, classification system, and mechanism of transduction followed by a summary of the existing literature on use of CPPs as gene delivery vectors either in the form of modified viruses, plasmid DNA, small interfering RNA, oligonucleotides, full-length genes, DNA origami or peptide nucleic acids.

## 1. Introduction

The plasma membrane of a cell is essential to its identity and survival, but at the same time presents a barrier to intracellular delivery of potentially diagnostic or therapeutic cargoes. Therefore, the development of approaches to deliver functional cargoes, be they peptides, proteins, nucleic acids, or nanoparticles across cell membranes, a process termed protein transduction, has wide-reaching research and clinical implications. The ability of Trans-Activator of Transcription (Tat) protein of the Human Immunodeficiency Virus (HIV) to transduce cultured cells and lead to viral gene expression [1,2] without requiring a receptor was the first example of a protein that naturally employs a portion of itself to achieve cell penetration and lead to intracellular delivery of the entire HIV viral particle. The chemical cross-linking of a full-length Tat protein to multiple different proteins such as horseradish peroxidase, ß-galactosidase, RNase A and domain III of Pseudomonas exotoxin A served to demonstrate the ability of Tat protein to ferry other large cargoes across the cell membrane [3]. Similarly, the homeobox Antennapedia (Antp) transcription factor of *Drosophila melanogaster* was demonstrated to enter nerve cells in a receptor independent manner where it could then regulate neural morphogenesis [4]. Mapping of the domains within Tat and Antp responsible for the observed transduction led to the identification of the first two cell penetrating peptides (CPPs): the 11 amino acid cationic domain of HIV-1 Tat protein (YGRKKRRQRRR) [5] and the 16 amino acid sequence from the third helix of the Antennapedia domain (RQIKIWFQNRRMKWKK) termed Antp or penetratin [6]. Subsequently, the ability of the small part of the full-length Tat protein to deliver cargoes, including other full length proteins and even large multimeric protein complexes across cell membranes in culture and in vivo following systemic delivery in mice [7] was documented, further highlighting the delivery potential of these unique peptides. Since then, the number of peptides, both cell-specific and non-specific, reported as having cell penetrating properties has increased exponentially [8]. This is particularly true for a wide spectrum of cationic peptides that primarily rely on their cationic charge to interact with proteoglycans on the cell surface (see below). There has been intense interest in identifying both new cell-specific CPPs, as well as strategies to make Tat and other non-specific CPPs act in a more cell-specific manner by taking advantage of tissue characteristics, mostly in the context of cancer. With the accompanying interest and explosion in the number of studies, it has become impossible to provide a comprehensive, all-encompassing single review of these unique peptides. Therefore, out of necessity, the authors chose to provide only a very broad overview of classification, mechanism of transduction and highlight applications of CPPs as vectors for gene and nucleic acid delivery only. Readers are referred to an earlier review of CPPs by the authors [8] as well as several other excellent reviews [9,10,11]. 

## 2. Types of CPPs

### 2.1. Non-Cell-Specific CPPs

CPPs are broadly categorized into non-cell-specific and cell-specific peptides with great sequence heterogeneity (Table 1). The non-cell-specific CPPs can be sub-classified into three classes: cationic, hydrophobic, and amphipathic. Tat and Antp are cationic peptides, rich in arginine and lysine, with the longer Antp peptide having a more defined 3D structure. In addition to these naturally occurring CPP sequences, synthetic cationic peptides including homopolymers of arginine [12], lysine [13] and/or the cationic, amino acid ornithine [14] were demonstrated to function as effective transduction peptides. Even histidine, which becomes protonated at low pH, can function as a CPP at pH below 6.0 and has been used for delivery into tumor cells with lower pH [15]. Arginine-based homopolymers ranging from 6 to 12 amino acids function as CPPs with 8–10 amino acid length identified as having the highest transduction ability [16]. Similarly, 8-mer homopolymers of lysine transduce a variety of cell types with similar efficiencies as homopolymers of arginine [17]. There is a definite optimum length for these homopolymers with greater than 12 amino acids showing reduced transduction efficiency. Unlike the acute cellular toxicity elicited by long poly-lysine molecules, short lysine homopolymers (6–12 mers) have no demonstrable cytotoxic effects, even at high concentrations [13]. Thus, it appears that too little or too much cationic charge within a short region negatively affects transduction. 

Amphipathic CPPs are chimeric peptides generated by attaching the hydrophobic domain of the CPP to a nuclear localizing signal (NLS) such as the SV40 NLS through a covalent bond [18]. Usually, hydrophobic CPPs are derived from signal peptide sequences. Signal peptides that allow proteins to be secreted from cells can also facilitate entry of the proteins back across the membrane into cells. Examples of hydrophobic transduction peptides identified to date include leader sequences for keratinocyte growth factor and fibroblast growth factor [19], but likely most leader sequences of secreted proteins could potentially function as CPPs.

Interestingly, even certain pathogenic bacteria use CPPs for delivery of bacterial effector proteins into different types of mammalian cells. For example, the pathogenic bacteria *Yersinia enterocolitica* encodes for the anti-inflammatory protein YopM with two alpha helices, α1H and α2H, in its amino terminus that function as CPPs similar to Antp [20,21,22]. Similarly, Shigella and Salmonella encode for immune effector proteins that can also enter cells efficiently to modulate the immune response [22].

### 2.2. Cell-Specific CPPs

The other major sub-classification is cell-specific CPPs, identified through different screening methods including plasmid, microorganism surface, ribosome, or phage display of large peptide libraries. The advantage of this approach is that a priori knowledge of a binding partner is not necessary. Such cell-specific CPPs circumvent the issues associated with the non-cell specific CPPs, namely non-specific cellular uptake leading to off-target side effects and the need to administer high doses of CPPs to achieve adequate levels in target organs or cell types of interest. Such high doses are necessary in order for a small fraction of CPPs to escape the liver, kidney, and the reticuloendothelial system to reach the target organ of interest. Such an approach would be particularly troublesome if the target is the brain or a poorly vascularized tissue. Thus, developing tissue or cell-specific CPPs would be particularly attractive as it would improve the efficacy of the delivered cargo with less off-target effects while reducing the overall dose needed, which would be particularly beneficial when scaling up from small to larger animal models, and ultimately for human clinical trials. 

Another approach to circumventing this issue is by delivering non-tissue specific CPP bearing cargo in a pro-drug fashion that can be activated under certain conditions or specific environments. This is only feasible if a specific cell type expresses a unique enzymatic activity such as viral or disease specific proteases [23]. Another approach is local delivery (e.g., intra-tumoral, intra-articular, intra-muscular, intra-ocular, intra-tracheal, intra-dermal, etc.) to limit the transduction activity of non-specific CPPs to specific sites. This would depend on and be limited to a specific application or situation and would be feasible only if the target cell is located in an accessible site with limited diffusion such as topical delivery for dermatological applications, the eye for ophthalmological applications [24,25,26], joints for arthritis/degenerative conditions [27], and directly into tumors [28]. 

## 3. Identification of Tissue Specific CPPs

Cell-specific CPPs were identified predominantly by using peptide phage display libraries to screen for peptides able to target specific cell types. The concept of phage display was first proposed by Smith in 1985 [29]. Following this initial report, combinatorial peptide libraries of various lengths using different types of phages (M13, T7) have been used successfully to identify peptides able to facilitate internalization of intact phage. Alternatively, plasmid, antibodies, microorganism surface or ribosome displays of peptide libraries have been employed as well. Phage display requires exposing the target cell or tissue of interest to a large, randomized phage library in which one of the envelope proteins used by the phage for internalization has been modified to display linear or cyclic peptides of various lengths and randomized amino acid sequences [30]. The internalized phage can then be isolated, expanded and used in subsequent rounds of screening. Usually 3–5 rounds of screening results in enrichment of a small number of peptides identifiable by DNA sequencing of the recovered phage. This approach requires enriching for a specific, small subset of phage from a very large library. As such, false positives are a concern and have to be discerned from phage that is indeed bound and internalized by the target cell type. One approach to circumvent this problem is to carry out the first cycle in cell culture using relevant cell types in order to reduce the likelihood of false positives. Subsequent cycles can then be carried out in vivo using the enriched pool from the in vitro cycle [30,31,32]. Such an approach has led to the identification of peptides targeting vascular endothelium [33], synovial tissue [27], dendritic cells [34], pancreatic islet cells [35] and cardiac myocytes [31], and has identified NRG (Asparginine-Arginine-Glycine) and RGD (Arginine-Glycine-Aspartic acid) motifs that target phage to tumor vasculature in nude tumor-bearing mice [36]. 

## 4. Mechanisms of Transduction

Despite intense study of CPPs, the specific pathway(s) involved in facilitating transduction remain elusive. CPPs are short in length, making the use of standard techniques for identifying binding targets on the cell surface more challenging. Additionally, their rapid cell entry, occurring within minutes at physiological conditions, makes analysis difficult. It is likely that a non-cell specific CPP such as Tat that crosses the blood brain barrier will not share a cell entry pathway with a cell-specific CPP. Even for a particular CPP the mechanism of transduction likely varies depending on the specific cargo fused to it, with changes in parameters such as local milieu and pH almost certainly playing a part. This is further complicated by recent data suggesting that the local concentration of a CPP may influence the internalization pathway used [37,38,39]. It should be noted that elucidation of the mechanism of transduction is not only of theoretical interest as the loading of cargoes must be achieved in a way as to not interfere with either the binding or cell internalization mechanism of CPPs. For example changing the hydrophobicity, but not the cationic charge, of a guanidine-rich homo-polymer significantly changed its transduction abilities and ability to internalize cargoes [40]. 

Although the exact transduction mechanism of CPPs remains elusive, extensive work from multiple investigators has shed considerable light. There is evidence both for mechanisms that are non-endocytic/energy independent or endocytic/energy-dependent [41]. The broad range of cells that are readily transducible by non-specific CPPs such as Tat suggests a role for ubiquitously shared cellular structures such as surface binding to plasma membrane phospholipids or, in particular proteoglycans through electrostatic interactions, as a first step towards cell entry [42,43]. Cell lines deficient in heparan sulfate have significantly reduced transduction by cationic peptides [13,44,45]. This reduction suggests that electrostatic interactions on the cell surface, separate from CPP-lipid interactions, contribute to protein transduction. It is likely that transduction is a two-step process, with the first step being electrostatic interaction of non-specific CPPs with anionic elements, such as glycosaminoglycans on the cell surface that draw the CPPs into close proximity to the plasma membrane. Subsequently, cationic CPPs bearing small cargoes likely enter cells via direct translocation, with uptake of larger cargoes mediated by micropinocytosis, a more energy-dependent and slower process [46]. Transduction has been shown to occur at 4 °C and after depletion of the adenosine triphosphate (ATP) pool [13], albeit at a reduced level, suggesting that it is not exclusively an energy-dependent process. Research also suggests that increasing hydrophobic characteristics of a CPP, as in the case of Tat, increases its efficiency as a transporter [47]. 

## 5. Cell Penetrating Peptides as Gene Delivery Vectors

Although CPPs have been used as vectors for delivery of drugs [48,49,50,51], other peptides of therapeutic potential [52,53,54,55,56,57,58,59,60], proteins [56,61,62,63,64,65], radioisotopes [66,67,68,69], quantum dots [70,71] and photosensitizers [72,73], this review will focus on use of CPPs as vectors for nucleic acid delivery, be they genes, oligonucleotides, peptide nucleic acid conjugates, small interfering RNA (siRNA) or the newest application with DNA origami. Although there is literature on all of these applications, most interest and work done to date has been with siRNA. The necessary factors to consider in these applications is the platform’s ability to successfully conjugate nucleic acids to CPPs, escape from enzymatic degradation in serum, escape the reticuloendothelial compartments, successfully cross cell membranes, escape from endocytic degradation and, in cases of gene delivery, achieve nuclear localization. All of these hurdles have been addressed with creative strategies that we will try to summarize here. Even though CPPs are attractive alternatives to viral gene delivery, we will begin by briefly summarizing the literature on use of CPPs to modify viral vectors for the purpose of gene therapy. 

### 5.1. Viral Vectors

Adenovirus is commonly employed as a vector for gene delivery due to its high transduction efficiency, although it is dependent on the presence of coxsackievirus and adenovirus receptors (CAR) for internalization. Chemical conjugation of adenovirus to Tat, octa-arginine or proline rich CPPs, led to 1–2 log-fold higher transduction of CAR-negative cells in vitro than unmodified adenovirus [74,75,76]. Electrostatic formation of complexes between negatively charged adenoviral particles and positively charged CPPs, such as Tat, penetratin, poly-arginine and Pep1, led to 100-fold greater transduction of CAR-negative cells compared to unmodified adenovirus, though again only in vitro [77]. In contrast, oncolytic adenovirus-serotype 5 modified to express an SCG3 promoter/ASH1 enhancer driven E1A gene expression, further modified to express Tat in the viral capsid protein, led to delays in tumor growth and prolonged survival of nude mice harboring subcutaneous human neuroblastoma xenografts [78]. In a very interesting application, brain-derived neurotrophic factor was fused with Tat and another CPP, HA2, and packaged into adeno-associated virus. Upon intranasal delivery to mice subjected to chronic mild stress, there was significant amelioration of depressive symptoms and increased hippocampal protein levels of brain-derived neurotrophic factor [79]. In another application, Tat was displayed on surface of MS2 bacteriophages carrying miRNA-122. Not only were there tumor inhibitory effects seen in multiple hepatocellular cancer cell lines, but also decreased tumor growth in nude mice bearing the hepatocellular cancer xenografts after they received an injection of the modified bacteriophage [80]. More recently, Sun and colleagues generated a recombinant PP7 bacteriophage using a one-plasmid double expression system to carry pre-microRNA-23b and displaying a CPP peptide that was able to inhibit hepatoma cell migration in vitro via down-regulation of liver-intestine cadherin expression [81]. Tobacco mosaic virus, a plant virus, decorated with Tat was able to carry enhanced green fluorescent protein (EGFP) silencing siRNA in vitro and to transduce EGFP expressing highly metastatic hepatocellular carcinoma cell lines in vivo in mice, with treatment for 10-days achieving over 80% EGFP-negative rates in tumors [82]. 

### 5.2. Plasmid DNA

As early as 2003, investigators realized the potential of delivering plasmid DNA using CPPs [83,84]. As Tat is rich in positively charged arginine and lysine residues, electrostatic interactions between Tat and negatively charged plasmid DNA allows for complexes to be formed between Luciferase or EGFP expressing plasmids and Tat. Though there was uptake in cells in vitro, it was via an endocytic pathway. Additionally, in vivo transfection rates were low with the internalization of complexes hindered by serum albumin [83]. To escape from endosomes after internalization, stearylation of CPPs has been used [85,86]. Stearyl-transportan 10 formed stable nano-complexes with plasmid DNA, was non-cytotoxic, non-immunogenic, and mediated transfer/expression of Luciferase reporter plasmid, though with only local uptake after an intra-dermal or intra-muscular injection [85]. Modifying another CPP, S4(13)-PV, with a 5 histidine residue attached to its N-terminus resulted in successful packaging of plasmid DNA and siRNA in vitro with successful gene silencing of Survivin as compared with the unmodified SV(13)-PV [87]. Poly-lysine homopolymers have been used to condense with plasmids bearing angiotensin type II receptor using calcium chloride. This approach produced negligible cytotoxicity in four different human cell lines, successful tumor targeting, and marked attenuation of lung cancer growth in vivo [88]. 

**Figure 1:**
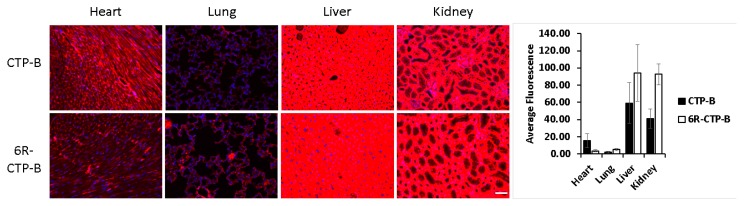
Fluorescent micrographs of heart, lung, liver and kidneys from mice euthanized 15 min after an intravenous injection with fluorescently labeled CTP-B or 6-Arginines-CTP-B. *N* = 3 for each peptide. Scale bar represents 50 μm.

Another approach used is one of dual targeting. This was achieved by synthesizing biotinylated forms of a tumor targeting peptide (CREKA) and a homo-polymer of arginine by linking them together through an avidin moiety. This complex could successfully condense with plasmid DNA carrying the p53 gene [89], with tumor regression seen in mice, but again the injections were adjacent to the tumor, bringing into question whether this reflected true targeting. Other homopolymers of arginine were synthesized with tumor targeting peptides [90] or a pro-apoptotic peptide (AVPI) [91] to enhance selective targeting of tumor cells. The latter conjugate was condensed with plasmid DNA carrying p53, to act synergistically to induce apoptosis in a multi-drug resistant breast cancer line, and indeed proved efficacious in vivo [91]. However, it is difficult to understand why these dual targeting complexes would specifically target tumors, as both poly-arginine and poly-lysine are non-specific CPPs. Excited by these reports, we constructed a tandem peptide by adding 6-arginine to the N-terminus of an optimized version of our cardiac targeting peptide (CTP-B). Based on our prior bio-distribution studies [66], this fluorescently labeled peptide was injected intravenously into mice and allowed to circulate for 15 min. However, the conjugate peptide actually reduced cardiac uptake compared to the parent CTP-B peptide, while increasing kidney and liver uptake (Figure 1).

### 5.3. Small Interfering RNA

Although CPPs have been used to deliver siRNA to the heart ex vivo [92,93], or for intradermal delivery [94,95], the vast majority of applications of this approach has been in the context of cancer therapeutics. The hurdles to successful siRNA delivery are similar to delivery of plasmid DNA, namely successful conjugation to CPPs, protection from RNAses in circulation, targeted delivery to organ or cancer of interest, successful uptake, and endosomal escape. Various, creative strategies have been employed to surmount these barriers. Electrostatic charge interaction between cationic CPPs and negatively charged siRNAs have produced self-assembly of CPPs and siRNA into nanoparticles [96,97,98,99]. Escape from endosomal compartments has been achieved by acylation [99], stearylation [100] or histidine modification [101] of the N-terminus of CPPs. Histidine is purported to act as a proton pump within lysosomal compartments leading to swelling, leakiness, and bursting of these compartments releasing the CPP/siRNA conjugate. 

In vivo targeting approaches have ranged from dual targeting to dual therapies. Tumor extracellular matrix has a high level of αvβ3/5 integrin expression that binds cyclic RGD peptide. Tandem peptides combing a CPP with tumor targeting RGD through a disulfide bond complexed with anti-KRAS siRNA formed nanoparticles that significantly delayed tumor growth in a mouse model of pancreatic cancer [102]. In a similar manner, a peptide targeting epidermal growth factor, overexpressed in oral cancer cells, was conjugated to an endosomal disruptive peptide, to successfully deliver siRNA targeting cancerous inhibitor of protein phosphatase 2A (CIP2A), an oncogene [103]. The association with the siRNA was through electrostatic interactions between the peptides and siRNA when mixed in a 60:30:1 ratio with successful silencing of CIP2A in vivo in mice bearing oral cancer tumor xenografts [103]. In another report, a tumor environment responsive nanoparticle was generated, comprised of a polyethylene glycol shell with a tumor pH-responsive polymer core. The core contained the tumor targeting RGD peptide along with homo-polymer of arginine associated through electrostatic interactions with siRNA silencing bromodomain 4. This construct was able to inhibit prostate cancer growth significantly [97]. 

Differences in tumor microenvironment, like lower pH, increased matrix metalloproteinase 2 expression and increased glutathione in the cytosol, have been employed to deliver siRNA using activatable CPPs to silence Rac1 in order to reduce hepatic metastases in colon cancer [104] and suppress c-MYC gene expression in breast cancer cell lines in vivo [105]. As anti-angiogenic therapy, stearylated poly-arginine was modified with histidine, loaded with anti-VEGF siRNA through electrostatic interaction, and Fausidil, a selective Rho-kinase inhibitor. In vitro and in vivo studies showed strong efficacy for cellular uptake and tumor growth inhibition [101]. 

### 5.4. Nanoparticles

As liposomes are a subset of nanoparticles (NPs), in this section we will focus on selected literature pertaining to non-lipid-based NPs modified in various ways with CPPs that showed efficacy in vivo. Targeting of NPs has been studied largely in the context of tumor therapies using Tat or homopolymers of arginine of various lengths. As both of these are non-cell specific CPPs, investigators have harnessed tumor environment specific properties, such as lower pH, increased matrix metalloproteinase 2 expression and increased redox potential, to make these non-specific CPPs act in a more tumor-specific manner. In one of the earliest studies, cationic NPs composed of β-cyclodextrin and low-molecular weight polyethylenimine were labeled with both octa-arginine and folic acid to deliver plasmid DNA to folate-receptor positive tumor cells, both in vitro and in vivo [106]. In another application gelatin-silica NPs were modified with different CPPs, including a fusogenic peptide comprised of Tat and influenza hemaglutanin A2, to successfully deliver plasmid DNA with endosomal escape and nuclear targeting properties in vivo [107]. A tumor activatable CPP dual-triggered by lowered pH and matrix metalloproteinase 2 was used to label NPs carrying dual anti-tumor therapies, doxorubicin and siRNA targeting vascular endothelial growth factor. This led to effective shut down of blood vessel formation and to apoptosis within the tumor [108]. In another approach, NPs were modified with photo- and pH-responsive CPPs. The cell penetrating ability of the cationic CPPs was quenched by a pH-sensitive, negatively charged inhibitory peptide that was released in the lower pH microenvironment of the tumor. These NPs, loaded with siRNA, accumulated within tumor cells upon near-infrared light illumination, resulting in increased antitumor efficacy in vivo [109]. Another approach employed was PEGylation of glycosaminoglycan-binding peptides coupled with DNA through electrostatic charge interactions, which formed NPs that targeted bronchial epithelial cell lines with precision cut lung slices in vitro showing that PEGylation rates of >40% were the optimal formulation [110]. Additionally, PEGylated supra-magnetic iron oxide NPs [111,112], Nobel metal NPs [113] or gold particles [114] have also been modified with CPPs to show internalization into tumor or stem cells. These latter studies were, however, all in vitro. 

### 5.5. Liposomes

Cationic liposomes (LP) form spontaneous complexes with nucleic acids, which can be sterically stabilized through PEGylation. One study explored the modification of such LPs with CPPs and found that full peptide coverage resulted in less internalization into cells than intermediate coverage, with optimum coverage being cell specific [115]. Additionally, cationic LPs, though effective in vitro, have demonstrated significant cytotoxicity in vivo. This issue can be addressed by using neutral PEGylated LPs modified by CPPs. In one study, neutral PEGylated LPs modified by octa-arginine homo-polymer showed negligible cytotoxicity, enhanced cellular association, and gene silencing capacity in vitro [116]. Harnessing the increased redox milieu of tumors, a Tat-functionalized LP was loaded with paclitaxel, a common cancer chemotherapeutic, with Tat conjugated to a pegylated (PEG) moiety through a cleavable disulfide linker. At the tumor site, PEG was detached by exogenous reducing agent glutathione, resulting in exposure of Tat, with subsequent enhanced tumor uptake and inhibition of proliferation of murine melanoma cell lines, both in vitro and in vivo [117]. In another approach, alanine-alanine-asparagine, a substrate for the endoprotease legumain, was added to the fourth lysine of Tat leading to a branched peptide version. This modified branching Tat peptide was used to label LPs loaded with doxorubicin, leading to decreased non-specific uptake and increased uptake by tumor cells, resulting in increase in anti-tumor activity and decrease in systemic side effects [118]. These data again demonstrate the ability to harness the cell penetrating capabilities of a non-specific CPP such as Tat in a tumor environment specific manner. 

### 5.6. DNA Origami

**Figure 2:**
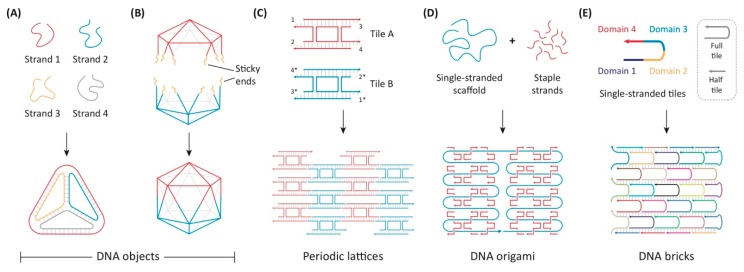
Structural DNA nanotechnology approaches for drug delivery. Common methods for creating DNA nanostructures include (**A**,**B**) junction and lattice-based structures like DNA nanocages, (**C**) periodic lattices, (**D**) scaffolded DNA origami and (**E**) scaffold-free assembly of single-stranded tiles (SSTs), otherwise known as DNA “DNA bricks”. Panels (A through E) reprinted with permission from Madhanagopal, B. R. et al. [129]. Copyright 2018, Elsevier.

**Figure 3:**
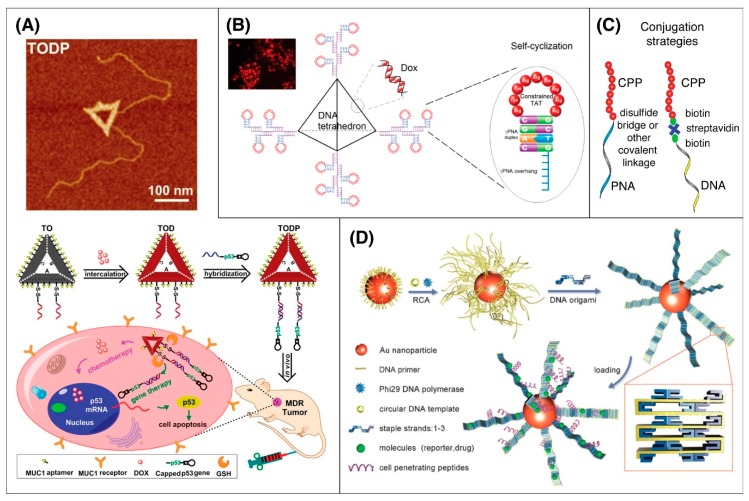
Drug delivery nanostructures can integrate multiple types of functional decoration and for cell-specific and high-efficiency transduction. (**A**) Decoration with MUC1 aptamers, DOX, and p53 gene cassettes enabled a DNA origami triangular construct to target tumor cells and delivery a combination therapy of chemotherapy agent DOX alongside p53 genes. Reprinted with permission from Liu, J. et al. [128]. Copyright 2018, American Chemical Society. (**B**) DNA tetrahedron structures were decorated with both DOX and Tat cell targeting peptides for targeted therapy. When Tat peptides were self-cyclized by the hybridization of short flanking γPNAs, the self-cyclized Tat constructs were taken up at 10-fold higher rates. Reprinted with permission from Tan, X. et al. [133]. Copyright 2018, American Chemical Society. (**C**) While CPP is typically conjugated to peptide nucleic acids (PNA) using disulfide bonds or other covalent methods, CPP is typically ionically bound to PNA using biotin-streptavidin attachment. (**D**) CPP-decorated DNA origami ribbons and gold nanoparticles were used together to create superstructures with high molecular loading capacity for both cellular imaging and drug delivery. Reprinted with permission from Yan, J. et al. [130]. Copyright 2015, John Wiley and Sons.

DNA nanostructures, first described by Nadrian Seeman [119], are novel structures formed by the hybridization of multiple single-stranded DNA (ssDNA) oligomers to create a variety of precise structures, with structure, size, and capabilities of DNA nanofabrication growing significantly in the intervening years (Figure 2A,B). In particular, the development of scaffolded DNA origami by Rothemund uses long ssDNA, typically from the M13 bacteriophage, in combination with hundreds of short ssDNAs that act as “staples” to form nanostructures with arbitrary shapes (Figure 2D) [120]. Approaches using only short ssDNA also demonstrated capability of forming two-dimensional [121,122] and three-dimensional structures (Figure 2C,E) [123,124]. Together, these methods treat DNA as a biopolymer, and its information-containing structure is used to drive nanostructure assembly. These DNA origami platforms can be decorated with biomolecules, including peptides, proteins, and functional molecules including fluorophores, aptamers, quantum dots, and gold nanoparticles, allowing them the ability to target cells [125,126]. In one example, the efficiency of intracellular delivery of DNA origami could be increased 22-fold when it was decorated with the iron transport protein transferrin, with efficiency increasing with increasing number of transferrin molecules attached [127]. The versatility of decoration of DNA origami has enabled the co-delivery of chemotherapeutics along with gene therapy. Liu et al. recently demonstrated the delivery of both doxorubicin and the p53 gene using a triangular DNA origami platform decorated with multiple targeting aptamers (Figure 3A) [128].

To date, only a few DNA nanotechnology studies used CPPs. One study used gold nanoparticles coated with CPP-decorated filamentous DNA origami to create a 3D superstructure with high transduction efficiency [130]. This approach demonstrated the versatility of DNA nanotechnology and its potential for cellular imaging and drug delivery [130]. Another multivalent approach for CPP and structural DNA nanotechnology used both aptamers and CPP to target Ramos cells and then drive the nanopore-like structure into the tumor cells [131]. The combination of aptamers with CPP in this study showed improved cellular uptake and targeting. DNA tetrahedrons have been used as vehicle for delivering siRNAs into cells to silence genes [132], and for the purpose of optimizing delivery, numerous targeting cationic and amphipathic CPPs including Tat, penetratin, and MAP were used. However, likely due to the overexpression of folate receptors in tumor membranes, decoration with folate as a targeting model produced more efficient uptake than platforms decorated with CPPs. In another application, a gammaPNA (γPNA) hairpin was used to create a non-covalently cyclized form of Tat to enhance cell uptake [133]; when DNA tetrahedra were used to increase the multi-valency of this approach, cellular delivery efficiency increased further (Figure 3B).

Like a “Swiss army knife” with many capabilities, DNA nanotechnology has the potential to create multivalent, dynamic and responsive gene delivery platforms capable of unprecedented control over targeting and delivery while integrating multiple distinct approaches using CPPs [134,135]. However, for optimization of this technology, robust and low-cost stabilization methods are critical for protecting DNA origami from low salt denaturation and enzymatic degradation in vivo.

### 5.7. Peptide Nucleic Acids

Peptide nucleic acids (PNAs) are peptide-like polymers with nucleic acid side chains. PNAs were first developed as a synthetic DNA mimic in which the deoxyribose phosphate backbone was replaced with an uncharged and achiral polyamide backbone [136]. With a similar axial rise and identical nucleobase side chains, PNAs are notable for their capacity to bind DNA with extraordinarily high affinity [137,138,139,140,141]. Furthermore, the pseudopeptide backbone of PNA is not a substrate for nucleases or proteases [137], giving them stability and potential to form nano-systems with “trojan horse”-like properties. Without modification, PNAs are not readily taken up by cells in vivo [142], but covalent conjugation to CPPs is straight-forward since both can be made using solid phase peptide synthesis (Figure 2C). In practice however, CPPs and PNAs are often separately synthesized and then conjugated via a disulfide bond [143]. Depending on the PNA nucleobase composition, they are capable of binding to complementary DNA and RNA via both Watson-Crick and Hoogsteen base pairing rules [144]. Important for cell delivery applications like gene therapy, covalent linkage to CPPs does not reduce the biological activity of uncharged PNA [142]. Once inside the cell, PNAs are well-suited for the steric blocking method of gene expression control, since they bind tightly to RNA and DNA while resisting degradation. 

The 16-residue CPP penetratin has most commonly been studied in PNA-CPP conjugates [145]. Notably, PNA-penetratin studies showed that the CPP does not interfere with the binding of PNA to target DNA [146], but later studies did find that cellular uptake of PNA-penetratin varies as a function of cell type, temperature and concentration [143]. The first demonstration of targeted delivery of CPP-coupled PNA came in 1998 when penetratin and transportan were covalently coupled to a 21mer PNA [147]. These conjugates showed efficient uptake in Bowes melanoma cells, binding to mRNA and blocking galanin gene expression to modify pain response. Another study demonstrated that PNA coupled to a retro-inverso delivery peptide were rapidly taken up by neurons, and that these antisense conjugates were able to depress the amount of target mRNA in culture [148]. Dragulescu-Andrasi et al. created cell-permeable guanidine-based PNA-oligoarginine analogues [149]. Gamma-position modifications with l-serine [150] and hydrophilic (R)-diethylene glycol “miniPEG” [141] were able to pre-organize these γPNAs into helical structures with higher binding affinity and higher solubility. Modification of PNA may also reduce the required dose of PNA in therapeutic applications, because such modification can decrease the rate of physiological clearance [151]. Endosomal escape remains a challenge for PNA-CPP conjugates post-endocytosis, which further necessitates high dose administration. To increase therapeutic efficacy, CPP-PNAs can be co-administered with chloroquine or Ca^2+^ to facilitate conjugate escape into the cytosol [152]. 

A strategy combining DNA nanotechnology, PNAs and CPPs to form NPs has shown promising results with integration of modified γPNAs further improving cellular uptake [153]. Specialized NPs using DNA nanotechnology offer unique opportunities for compactly integrating precise ratios and arrangements of distinct functional molecules such as PNA and CPP onto DNA nanostructures. As mentioned in the previous section, one such approach was a DNA tetrahedron-based beacon decorated with Tat flanked by short complementary γPNAs (Figure 3B) [133]. In this study, as the complementary γPNAs hybridized, they formed a hairpin causing the CPP to “self-cyclize” to form into a conformationally constrained and higher activity state. Uptake studies of the beacon system showed a 10-fold increase in uptake of self-cyclized Tat-PNA systems as compared with linear ones. 

### 5.8. CRISPR-Cas

CRISPR-Cas (clustered, regularly interspaced, short palindromic repeats-associated system) represents an efficient tool for gene editing and consists of a guide RNA and the Cas9 protein delivered to cells using either plasmid or virus-based vectors. However, using CPPs, direct delivery leads to less cellular toxicity, and fewer off-target mutations [154,155,156] as well as delivery to hard-to-transfect cell lines [157]. Protocols were optimized to use this approach [158]. 

## 6. Hurdles to Clinical Application 

Few clinical trials using CPPs have been published to date, mostly in the context of cancer diagnosis and therapeutics [159,160,161]. Twenty-seven patients with breast cancer received intra-operative activatable fluorescent peptide to improve tumor margin detection and assist with complete tumor resection [162]. Intraoperative imaging of surgical specimens allowed for real-time tumor detection and tumor-free margin resection [162]. Another small trial of 31 patients undergoing coronary artery angioplasty used the cell penetrating lipopeptide protease-activated receptor-1-based pepducin as an anti-platelet agent and this approach was demonstrated to be safe [163]. However, the trial was too small to assess whether this offered superior therapeutic outcomes over standard therapies.

AVI-5126 is a CPP conjugated to a morpholino designed to knock-down the human C-MYC gene. It was the first CPP-conjugated morpholino tested in a safety and efficacy clinical trial to prevent blockage of veins harvested for cardiovascular bypass surgery by inhibiting cell proliferation. The vein was excised and immersed in a solution containing 10 mM AVI-5126 and then re-implanted as a bypass graft. The study, titled “Clinical Study to Assess the Safety and Efficacy of ex-Vivo Vein Graft Exposure to AVI-5126 in Coronary Artery By-Pass Grafting to Reduce Clinical Graft Failure”, was terminated early, not due to safety concerns, but due to low likelihood of clinical efficacy. The same CPP and morpholino conjugate were also tested in a restenosis of coronary artery after balloon angioplasty trial, enrolling 30 patients, but it was terminated for unknown reasons with no safety or efficacy data published. It is possible that the advent of drug-eluting stents and their remarkable efficacy has reduced the interest in the clinical development of this conjugate. 

Give the small number of clinical trials with CPPs, there clearly are significant issues to overcome before clinical use of CPP-based therapeutics becomes a reality. The first issue is the potential immunogenicity of the CPP containing protein or peptide, especially for chronic or repetitive treatments. It is unlikely that the small CPPs themselves would be immunogenic, but their cargoes can vary in size and the novel antigenic epitopes generated by fusion with the CPP could theoretically pose an issue. The second issue is lack of oral bioavailability of CPPs linked to their cargoes, and hence need for either topical or intravenous administration. One exception may be cyclized arginine rich CPPs with d- or l-naphthylalanine that showed some evidence of oral bioavailability with increased endosomal escape and improved cytosolic delivery [164]. The third issue, as discussed above, is the lack of cell specificity for the cationic and hydrophobic CPPs. The lack of specificity decreases the therapeutic window, increasing the dose used and hence potential for adverse effects. Fourth, since the two major routes of elimination for CPPs are the kidney and liver, analysis of toxicity of a CPP-based therapeutic in these and other tissues is required by the FDA prior to initiation of clinical trials. The testing of CPP-based therapeutics has to be carried out under Good Laboratory Practices (GLP) conditions and pharmacology/toxicity analyses by a GLP certified lab can be prohibitively expensive, with few federal, non-private funding mechanisms available. Lastly, CPP-based therapeutics will likely be expensive to produce and the cost of the therapeutic could be an issue, especially when treating a chronic condition that requires frequent dosing for extended time-periods. As a reference, it is estimated to typically take ~2.6 billion US dollars to bring a therapeutic to market. However, depending on efficacy and targeting, especially for cancers where therapies are time-limited, the cost-benefit ratio could be very favorable. For other conditions, delivering cargoes that last a few weeks or months, like siRNA, could reduce the frequency of administration, and potentially reduce treatment costs. 

## 7. Summary

The identification of cell penetrating peptides (CPP) or protein transduction domains (PTD) a quarter of a century ago has opened up new avenues to deliver peptides, proteins, nucleic acids, and nanoparticles, including viral particles, more efficiently into cells. Tissue specific CPPs, as well as non-specific CPPs that were engineered to target certain cell types, show great potential for clinical use. Their utility in diagnostic imaging is already being realized in the arena of tumor imaging. Despite the myriad positive results using CPPs in pre-clinical models for diagnosis and/or treatment of disease, the clinical applications of CPPs have been slow to develop due to the many hurdles to implementing new therapeutics. 

## 8. Patents

M.Z. and Paul D. Robbins (University of Minnesota, Minnesota, MN, USA) hold a patent on the use of cardiac targeting peptide as a cardiac vector (Cardiac-specific protein targeting domain, U.S. Patent Serial No. 9249184). 

## Figures and Tables

**Table 1:** Classification of Cell Penetrating Peptides.

**CPPs-Non-Tissue Specific**	**Peptide Sequence**	**Origin**
Cationic		
Tat [5]	GRKKRRQRRRPPQ	HIV Tat Protein
Ant [6]	RQIKIWFQNRRMKWKK	Antennapedia homeodomain
8-Arginine [12]	RRRRRRRR	n/a
8-Lysine [13]	KKKKKKKK	n/a
PTD-5 [17]	RRQRRTSKLMKR	Phage display
Hydrophobic		
Transportan [165]	GWTLNSAGYLLGKINLKALAALAKKIL	Galanin and mastoparan
MAP [166]	KLALKLALKALKAALKLA	Galanin and mastoparan
TP10 [167]	AGYLLGKINLKALAALAKKIL	
Pep-7 [168]	SDLWEMMMVSLACQY	CHL8 peptide
Amphipathic		
Azurin p18 [169]	LSTAADMQGVVTDGMASG	Azurin
Azurin p28 [170]	LSTAADMQGVVTDGMASGLDKDYLKPDD	Azurin
hCT18-32 [171]	KFHTFPQTAIGVGAP	Calcitonin
Bac 7 [172]	RRIRPRPPRLPRPRPRPLPFPRPG	Bactenecin
**CPPs-Tissue Specific**	**Peptide Sequence**	**Origin**
CTP [31,66]	APWHLSSQYSRT	Phage display
K5-FGF [173]	AAVALLPAVLLALLP	Phage display
HAP-1 [27]	SFHQFARATLAS	Phage display
293P-1 [174]	SNNNVRPIHIWP	Phage display
Vascular Endothelium [33]	SIGYPLP	Phage display
